# Case Report: A case of acute kidney injury due to star fruit-infused liquor

**DOI:** 10.3389/fmed.2026.1932834

**Published:** 2026-08-25

**Authors:** Sichun Shan, Miao Shen, Jiajun Shi, Wei Liu

**Affiliations:** Deqing People’s Hospital (Sir Run Run Shaw Hospital, School of Medicine, Zhejiang University), Zhejiang, Huzhou, China

**Keywords:** acute kidney injury, calcium oxalate, crystalline nephropathy, kidney biopsy, star fruit wine

## Abstract

Oxalate nephropathy (ON) is a syndrome characterized by renal dysfunction accompanied by the deposition of calcium oxalate crystals within the renal tubules. We report a rare case of secondary ON in a patient who developed acute kidney injury (AKI) following the consumption of large quantities of star fruit-infused liquor. The patient’s clinical presentation was unremarkable; however, laboratory investigations revealed a rapid elevation in serum creatinine levels over a short period, meeting the clinical criteria for AKI. Initial diagnosis was challenging. After a thorough review of the medical history and kidney biopsy, a definitive diagnosis of secondary ON caused by oxalate deposition of star fruit origin was made. Following active treatment, the patient’s renal function gradually improved, but long-term follow-up indicated progression to chronic kidney disease (CKD). This report aims to underscore the importance of clinical recognition of secondary ON, emphasizing that early identification of its etiology is crucial for the prevention of subsequent renal injury.

## 1 Introduction

Oxalate nephropathy (ON) is a disease characterized by the extensive deposition of calcium oxalate crystals, leading to tubular damage, interstitial fibrosis, and subsequent impairment of renal function (1). Based on etiology, ON is classified into primary and secondary types. Primary ON is primarily caused by genetic disorders of glyoxylate metabolism. Secondary ON typically refers to oxalate deposition secondary to other diseases, with the most common cause being enteric hyperoxaluria (2). Early diagnosis is critical due to the risk of severe renal impairment; however, the non-specific clinical presentation of the disease often results in underdiagnosis. Definitive diagnosis ultimately requires renal biopsy (3, 4). Apart from conventional approaches such as dietary modification and fluid supplementation, the efficacy of corticosteroid use for this condition remains inconclusive (5). ON may represent an underrecognized cause of renal failure, frequently manifesting as an acute exacerbation of chronic kidney disease (CKD) or acute kidney injury (AKI). The prognosis is poor, with a high occurrence rate of renal failure following diagnosis (6, 7). Although previous case reports have documented AKI following the ingestion of star fruit, its juice, or dried fruit, to the best of our knowledge, cases following the consumption of star fruit-infused liquor are rarely reported (8). This article provides a detailed description of the diagnosis, treatment course, and prognosis of this case.

## 2 Case report

A 44-year-old middle-aged male patient with no prior medical history was admitted to the hospital on April 10, 2025, with a chief complaint of fever that began 3 days before admission. The fever was predominantly low-grade, accompanied by poor appetite, nausea, and lumbar soreness. Laboratory tests performed on the day of admission revealed abnormal renal function. Physical examination showed stable vital signs, a pain score of 2, and auscultation revealed coarse breath sounds in both lungs. The abdomen was symmetrical and non-tender, bilateral renal percussion pain was negative, and there was no edema in the lower extremities.

Laboratory tests revealed abnormally elevated renal function parameters: urea (11.81 mmol/L) and serum creatinine (393 μmol/L). Blood gas analysis indicated metabolic acidosis (pH: 7.31). Urine sensitive renal function panel (seven items) showed elevated β_2_-microglobulin levels. Notably, the patient had no prior history of kidney disease, and this episode of AKI primarily involved tubular damage.

Further investigations, including determination of anti-streptolysin O (ASO), erythrocyte sedimentation rate (ESR), glycated hemoglobin (HbA1c), coagulation function tests, serum protein electrophoresis, hepatitis B serological markers (three-item panel), infection screening (three-item panel), complete antinuclear antibody (ANA) panel, anti-glomerular basement membrane (anti-GBM) antibody determination, and vasculitis-associated nephritis panel (four-item panel), were all negative. Electrocardiography, renal vascular ultrasound, and whole-abdominal computed tomography (CT) revealed no significant abnormalities. Detailed laboratory findings are presented in Supplementary Tables S1–S4.

During hospitalization, dynamic monitoring of renal function indicated progressive deterioration. On April 11, 2025, laboratory measurements revealed urea (13.82 mmol/L) and serum creatinine (540 μmol/L), demonstrating rapid progression of renal impairment. However, the underlying etiology remained unidentified. Laboratory findings suggested predominantly tubular injury; therefore, acute interstitial nephritis (AIN) was strongly suspected. Empiric anti-inflammatory therapy was initiated with intravenous methylprednisolone sodium succinate at a dose of 40 mg, supplemented with symptomatic treatments including urine alkalinization and fluid resuscitation (9). On April 12, 2025, repeat assessment of renal function showed a slight decrease in serum creatinine (473 μmol/L), suggesting that glucocorticoid therapy and supportive care could potentially control disease progression. Accordingly, in addition to routine supportive management, glucocorticoid therapy was continued (intravenous methylprednisolone sodium succinate 40 mg from April 11 to 13, 2025; renal function was re-evaluated on April 14; oral methylprednisolone 20 mg once daily from April 15 to 18, 2025).

During repeated inquiries into the patient’s medical history, it was found that the patient had steeped star fruit in white liquor at a ratio of approximately four fruits per 500 g in liquor for about 1 month. Four days before admission, approximately 500 mL of this preparation was consumed, and the aforementioned symptoms developed the following day. At that time, the intake of other high-oxalate foods, such as nuts and spinach, was denied.

Follow-up renal function tests on April 14, 2025, indicated partial recovery compared to previous levels; however, the recovery was slow, and the values had not yet returned to the normal range (serum creatinine: 417 μmol/L). Therefore, a percutaneous renal biopsy was performed on April 16, 2025. After 1 week of treatment, the patient’s body temperature returned to normal, appetite improved, and symptoms such as nausea and vomiting resolved. Renal function showed significant improvement compared to previous assessments, with a serum creatinine level of 293 μmol/L. The patient was discharged with medications on April 18, 2025 (methylprednisolone 20 mg once daily + sodium bicarbonate tablets 0.5 g twice daily).

**Figure 1:**
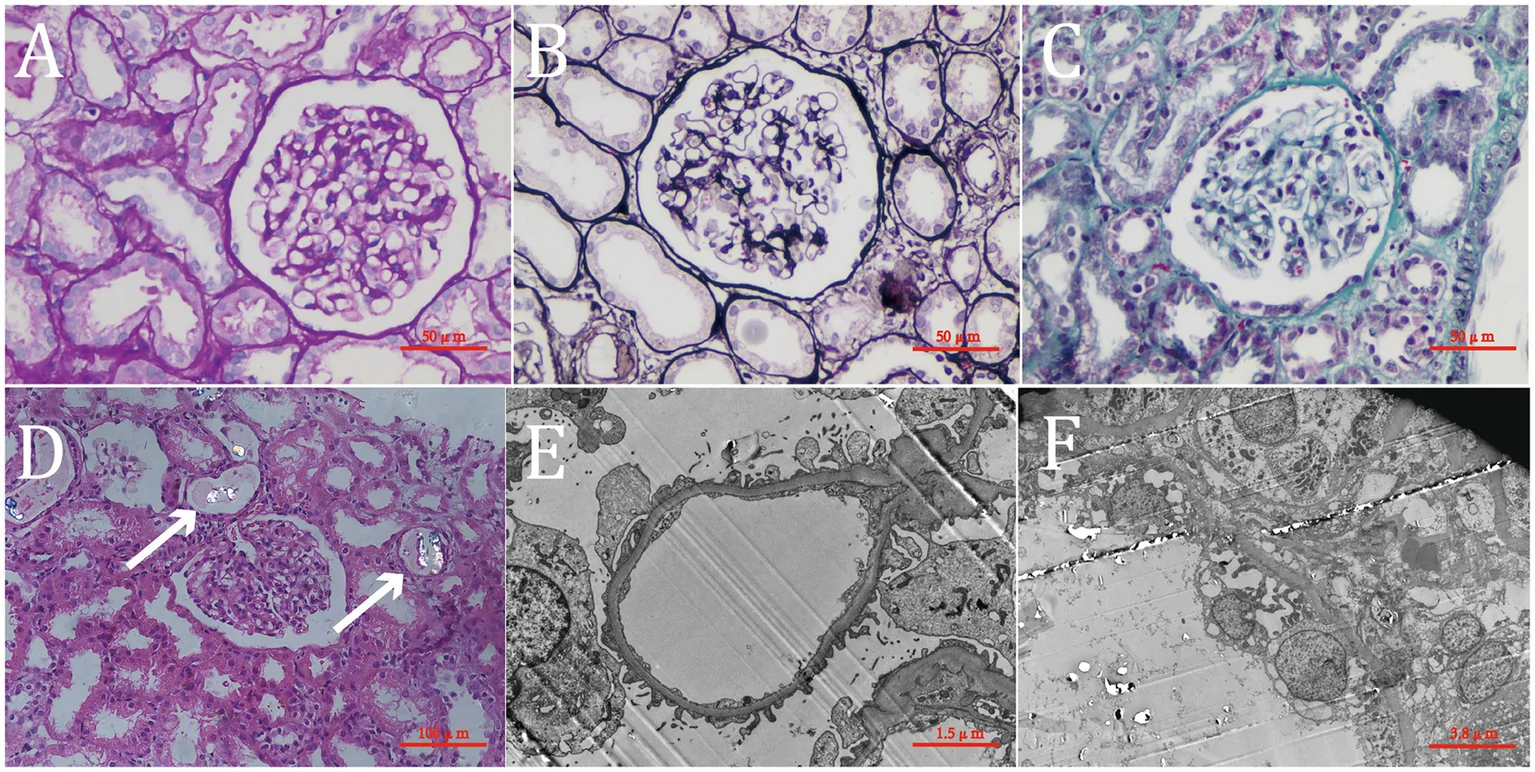
**(A)** Periodic acid–Schiff (PAS) staining, original magnification × 400. **(B)** Periodic acid–silver methenamine (PASM) staining, original magnification ×400. **(C)** Masson’s trichrome staining, original magnification × 400. **(D)** Hematoxylin and eosin (HE) staining under polarized light, original magnification × 200. Birefringent calcium oxalate crystals (white arrows) are visible within tubular lumina, confirming their presence. Microscopic description of renal tissue pathology report for panels **(A–D)**: A total of 25 glomeruli were identified in all sections. Glomerular changes were mild; capillary loops were well opened; no obvious swelling or hyperplasia of endothelial cells was observed; the mesangial regions showed no apparent alterations; and there was no segmental sclerosis, capsular adhesions, crescent formation, or necrosis. On PASM staining, the glomerular basilar membrane did not exhibit “spike,” “double contour,” or “chain-like” structures. Swelling of visceral epithelial cells (podocytes) was noted, together with segmental thickening and focal stratification of Bowman’s capsule. The tubular epithelial cells displayed granular and vacuolar degeneration. Focal sloughing of tubular epithelial cells and loss of the brush border were seen, with occasional exposure of the basilar membrane. A small proportion of tubular epithelial cells were flattened, and the tubular lumina were dilated. Occasional protein casts were present. Crystalline material was observed within some tubular lumina, which was birefringent under polarized light. Occasional tubular atrophy was found. In addition, focal (2%) interstitial edema accompanied by infiltration of a small number of lymphocytes and monocytes was present. Hyalinosis of small arteries and arterioles was occasionally observed. **(E)** Transmission electron microscopy, original magnification × 13,000. **(F)** Transmission electron microscopy, original magnification × 5,300. Electron microscopy of renal tissue for panels **(E,F)**: One glomerulus was examined. Glomerular changes were mild. Capillary loops were well opened, and no endothelial cell proliferation was seen. The mesangial areas were unremarkable, and no electron-dense deposits were detected in the mesangial, subepithelial, or subendothelial regions. No other special structures were observed. The basilar membrane was of normal thickness, with an average thickness of 320 nm. Segmental (<50%) foot process effacement was observed. Tubular epithelial cells showed mitochondrial swelling and dilatation of the endoplasmic reticulum. A few tubular epithelial cells were flattened with widened lumina. Focal loss of microvilli from tubular epithelial cells was noted. Thickening of the tubular basilar membrane was observed in a few tubules. A small number of lymphocytes and monocytes were seen in the interstitium. Arterioles were not identified.

The renal biopsy pathology report was returned on April 23, 2025. Light microscopy revealed mild glomerular alterations, whereas moderate tubular injury was observed. Under polarized light, birefringent crystals were visible within some renal tubules, suggestive of focal calcium oxalate deposition. Interstitial inflammation was mild and focal, involving approximately 2% of the cortical area and accompanied by scant lymphomonocytic infiltration. Occasional tubular atrophy was noted. Electron microscopy of renal tubular epithelial cells demonstrated mitochondrial swelling, dilation of the endoplasmic reticulum, loss of microvilli, and focal basilar membrane thickening. Immunofluorescence staining for IgA, IgG, IgM, C1q, C3, Fib, kappa, lambda, C4, IgG1, IgG2, IgG3, IgG4, PLA2R, and THSD7A was uniformly negative. On immunohistochemistry, only scattered interstitial cells positive for CD3 and CD20 were identified; staining for CD38, CD68, and MPO was negative. The specific results are presented in Figure 1. By integrating the clinical history, laboratory tests, and pathological findings, a diagnosis of ON was established, characterized by moderate acute tubular injury with focal crystal deposition and mild secondary interstitial inflammation.

After discharge, the patient was followed up regularly. In addition to sodium bicarbonate tablets (0.5 g twice daily), the dose of oral methylprednisolone was tapered according to medical advice until discontinuation (10 mg on May 2, 2025; 5 mg on May 16, 2025; and discontinued on May 23, 2025). On May 27, 2025, the patient’s serum creatinine level was measured at 142 μmol/L, and renal function had not yet fully recovered. The detailed clinical visit flowchart is shown in Supplementary Figure S1.

Thereafter, the patient returned to their hometown, and follow-up was conducted via telephone. The patient reported attending regular follow-up visits every 3 months at the Department of Nephrology, General Hospital of the Eastern Theater Command of the People’s Liberation Army. The most recent visit took place on June 21, 2026, at which time the serum creatinine was rechecked and found to be 121.11 μmol/L, remaining persistently above the upper limit of normal. Based on the results of multiple post-discharge follow-up assessments, the patient’s renal function never returned to normal, indicating that the condition had progressed from AKI to CKD.

## 3 Discussion

**Figure 2:**
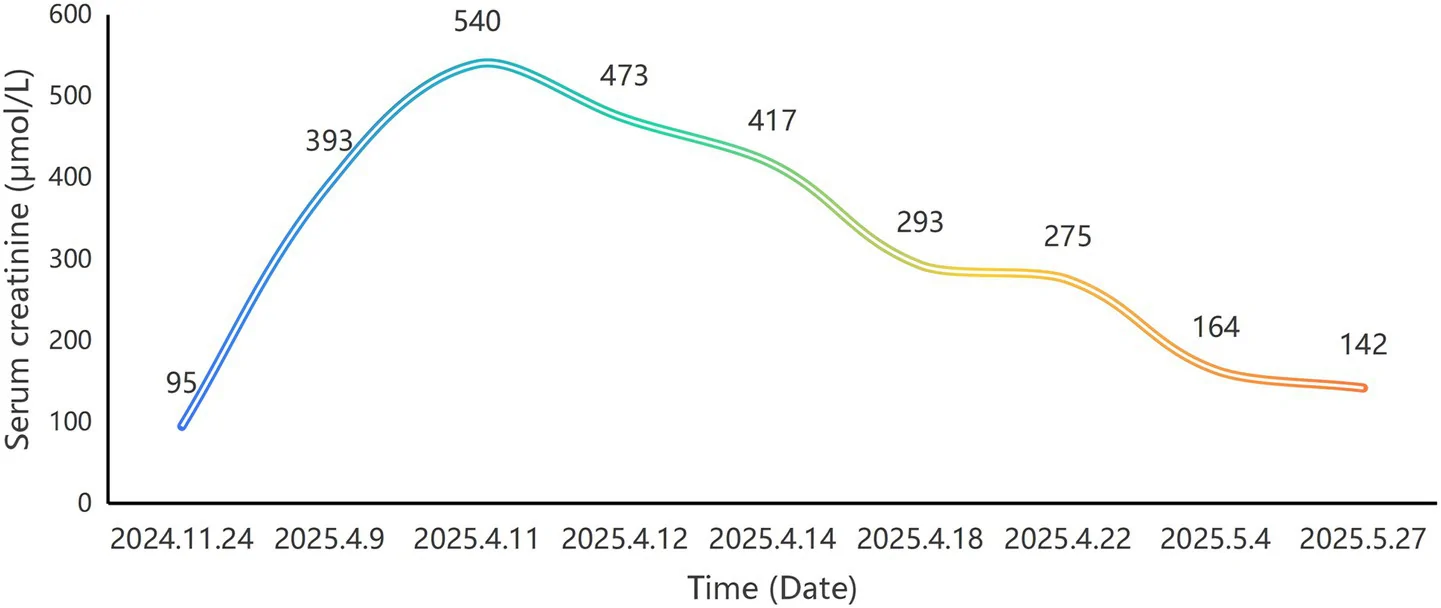
A dynamic monitoring linear chart of serum creatinine is presented; the reference interval for serum creatinine at our hospital is 57–97 μmol/L.

ON is a rare causative factor of AKI (10). Due to limitations in renal biopsy indications and tissue sampling, epidemiological data on ON are still lacking in China. Although case reports of ON are relatively rare worldwide, its actual incidence is not low (7). Therefore, for patients with CKD or AKI presenting with unexplained rapid deterioration of renal function, the possibility of secondary ON should be carefully considered (11). The case reported herein is one of secondary ON induced by excessive consumption of star fruit-infused liquor. During the diagnostic and therapeutic process, this otherwise healthy male with no prior history of kidney disease was initially diagnosed with AIN (with tubular injury) based on laboratory findings. In response to the rapid decline in renal function, empirical immunosuppressive therapy with corticosteroids was administered. However, the initial treatment response was poor, highlighting the current clinical challenges in the early diagnosis and limited pharmacotherapeutic options for this disease (Figure 2).

Primary hyperoxaluria is a relatively rare condition (12), whereas ON predominantly presents in a subsequent form. In clinical practice (13), enteric hyperoxaluria accounts for 60% of secondary ON cases (e.g., chronic pancreatitis or post-gastrointestinal surgery states) (14, 15), and excessive intake of oxalate-rich foods or their precursors accounts for 23% (e.g., spinach, nuts, and vitamin C) (16–18). Star fruit is also an oxalate-rich fruit (19). Following the ingestion of a large volume of star fruit-infused liquor, this patient developed an acute illness with a short clinical course, had no history of gastrointestinal surgery or malabsorption, and should therefore be unequivocally classified as a case of excessive oxalate intake. Meanwhile, it is speculated that heavy alcohol consumption over a short period exacerbated the renal dysfunction (20).

Differentiation from various other forms of AKI is required. The patient was previously healthy and denied any medication use or exposure to unknown substances before admission, thereby ruling out toxic nephropathy (21). Acute tubular necrosis (ATN) typically occurs in patients with a history of ischemia or toxin exposure, and urinary sediment generally reveals renal tubular epithelial cell casts or granular casts (22). In AIN, renal biopsy demonstrates interstitial infiltration of inflammatory cells (e.g., lymphocytes and eosinophils), and a history of drug allergy is often present (23). Infection-associated AKI is usually accompanied by a well-defined infection history and positive urine cultures (24). In uric acid nephropathy, urinary crystals are composed of urate, which displays negative birefringence under polarized light; in paraffin sections, only empty clefts left by dissolved crystals are frequently observed. This condition is associated with hyperuricemia and a history of gout (25). Cystinosis is mostly a hereditary finding (26). By reviewing the patient’s history, clinical manifestations, laboratory examinations, and renal biopsy features in this case, these differential diagnoses were sequentially excluded, and the disease was confirmed.

The mechanisms underlying oxalate-induced kidney damage have not yet been fully elucidated. The key pathophysiology may lie in the deposition of calcium oxalate crystals and the ensuing inflammation. When the oxalate load exceeds the renal excretory capacity, calcium oxalate crystals are formed through binding of oxalate to calcium ions. These crystals initially aggregate within the tubular lumen, leading to luminal obstruction and injury to tubular epithelial cells (27). It has been demonstrated that kidney damage can be induced by calcium oxalate crystals via multiple pathways. On the one hand, the crystals cause direct physical damage to renal tubular epithelial cells, resulting in cellular degeneration, apoptosis, and necrosis, with focal denudation of the tubular basilar membrane (28). This is consistent with the pathological findings observed in the present case. In some severe cases, calcium oxalate crystal-induced tubular injury can lead to tubular atrophy and sloughing, accompanied by interstitial fibrosis (29). In this case, tubular atrophy was observed occasionally, consistent with moderate injury. On the other hand, oxidative stress is activated in tubular epithelial cells, causing the generation of abundant reactive oxygen species (ROS) that further exacerbate cellular injury (30). In addition, intrarenal inflammatory pathways, such as the NLRP3 inflammasome, can be activated by calcium oxalate crystals, prompting the release of IL-1β and IL-18, which in turn recruit macrophages and neutrophils and establish an inflammatory microenvironment (31, 32). In the present case, the extent of inflammation was 2%, localized, and the infiltration was relatively mild, which may correspond to the focal rather than diffuse distribution of crystal deposition.

Currently, therapeutic options for secondary ON are limited and include a low-oxalate diet (recommended dietary oxalate intake of less than 100 mg/day), increased fluid intake, oral calcium supplementation (normal dietary calcium intake of 1,000–1,200 mg/day), and urine alkalinization using crystal inhibitors such as potassium citrate. Depending on the actual extent of kidney damage, renal replacement therapy may be considered (33). In some patients, renal biopsy primarily reveals oxalate crystal deposition and tubular injury, with relatively mild interstitial inflammation. In such cases, the value of anti-inflammatory therapy may be limited, and excessive use of corticosteroids could instead lead to immunosuppression and metabolic complications (34, 35). Fortunately, current preclinical studies have demonstrated significant potential for therapeutic strategies such as SIRT1 agonists, HDAC6 inhibitors, and exogenous itaconate supplementation (31, 36). These approaches point toward the development of targeted therapies for ON. Future research should aim to further elucidate these mechanisms to enable early diagnosis and more effective clinical intervention.

Several limitations should be acknowledged. First, quantitative measurement of urinary oxalate excretion (24-h or random urine), determination of serum oxalate concentration, and microscopic examination of urinary sediment for calcium oxalate crystals were not performed, because these specific tests are not included in the routine test menu of our clinical laboratory. The absence of these data precludes accurate assessment of the systemic oxalate burden and the degree of crystalluria (37). Future case reports incorporating such biochemical and microscopic evaluations would help to more completely elucidate the underlying pathophysiological mechanisms. Second, because glucocorticoids were administered empirically based on a presumptive clinical diagnosis of AIN before the diagnosis was confirmed by renal biopsy, it is not possible to precisely distinguish the extent to which the improvement in renal function was attributable to corticosteroid therapy versus supportive care and removal of the etiologic factor. We acknowledge that the evidence supporting corticosteroid therapy specifically for ON remains limited and inconclusive, and the mild (2%) interstitial inflammation does not strongly support continued immunosuppressive therapy. This limitation reflects the real-world challenge of empirical treatment when the diagnosis remains uncertain and further underscores the importance of performing renal biopsy early to establish a definitive diagnosis in similar clinical settings (38). Third, although the patient was followed up regularly at the General Hospital of Eastern Theater Command after discharge, complete follow-up data (such as quantitative urine protein, dynamic eGFR curves, and renal ultrasound) could not be directly obtained by our hospital. Therefore, only changes in serum creatinine can be reported, precluding a comprehensive assessment of CKD staging. However, the persistently impaired renal function over the long term (serum creatinine 142 μmol/L at discharge and 121.11 μmol/L at the last follow-up) unequivocally demonstrates progression from AKI to CKD. This finding carries profound clinical cautionary significance.

## 4 Conclusion

This case report describes a patient who developed AKI after consuming star fruit-infused liquor. The underlying pathophysiology is primarily attributed to the deposition of oxalate crystals within the renal tubules, leading to tubular injury and interstitial inflammation. The observation of birefringent crystals under polarized light microscopy, in conjunction with the patient’s history, can serve as a diagnostic criterion. Long-term follow-up further confirmed that, despite aggressive treatment during the acute phase, the patient still progressed to CKD. Therefore, early recognition, timely identification and elimination of the causative factor, provision of supportive care, and appropriate follow-up remain the core components of patient management.

## Data Availability

The original contributions presented in the study are included in the article/Supplementary material, further inquiries can be directed to the corresponding authors.
