# Supplementary material for: Case Report: A case of acute kidney injury due to star fruit-infused liquor
Source: Front Med (Lausanne). 2026 Aug 25;13:1932834. doi: 10.3389/fmed.2026.1932834 (PMC13549894; doi:10.3389/fmed.2026.1932834)
Supplement: Supplementary file 1 [file Supplementary_file_1.docx]

Supplementary Material

Supplementary Figures and Tables

## Supplementary Table S1-4: Detailed laboratory findings

Table S1 Renal function according to the evaluation time

| **Time (days)** | **Serum creatinine** | **Ref**  **(μmol/L)** | **Serum urea** | **Ref**  **(mmol/L)** |
| --- | --- | --- | --- | --- |
| **-136** | 95 | 57-97 | 4.33 | 3.10-8.00 |
| **1*** | 393 | ↑ | 11.81 | ↑ |
| **2** | 540 | ↑ | 13.82 | ↑ |
| **3** | 473 | ↑ | 13.08 | ↑ |
| **5** | 417 | ↑ | 14.59 | ↑ |
| **9** | 293 | ↑ | 8.89 | ↑ |
| **13** | 275 | ↑ | 8.29 | ↑ |
| **25** | 164 | ↑ | 5.80 | - |
| **48** | 142 | ↑ | 5.58 | - |
| **Note:*First evaluation day.** | | | | |

Table S2 Laboratory tests results

| **Blood test** | **Results** | **Ref** | **Urinalysis** | **Results** | **Ref** |
| --- | --- | --- | --- | --- | --- |
| Sodium (mmol/L) | 141 | 137-147 | Urine pH | 6 | 4.5-8.0 |
| Potassium (mmol/L) | 5.26 | 3.50-5.30 | Urine nitrite | Negative | Negative |
| Chloride (mmol/L) | 109 | 99-110 | Urine sugar | Negative | Negative |
| Total calcium (mmol/L) | 2.16 | 2.11-2.52 | Urine specific gravity | 1.01 | 1.003-1.030 |
| Phosphate (mmol/L) | 1.24 | 0.85-1.51 | Urine bilirubin | Negative | Negative |
| Uric acid (μmol/L) | 557 | 202-416 | Urine ketones | Negative | Negative |
| Total bilirubin (μmol/L) | 9.7 | ＜26.0 | Urine protein | Negative | Negative |
| Albumin (g/L) | 39.9 | 40.0-55.0 | Urine hemoglobin | Negative | Negative |
| AST (U/L) | 12 | 15-45 | Urine white blood cells | Negative | Negative |
| ALT (U/L) | 6 | 9-60 | Vitamin C | 2 | Negative |
| Total cholesterol (mmol/L) | 2.7 | ＜5.2 | Urine color | Pale yellow |  |
| IgG (g/L) | 12.41 | 6.50-16.00 | Red blood cells (cells/μl) | 0 | 0-17 |
| IgA (g/L) | 1.93 | 0.40-3.50 | White blood cells (cells/μl) | 1 | 0-28 |
| IgM (g/L) | 0.98 | 0.50-3.00 | Pathological casts (cells/μl) | 0 | 0 |
| Complement C3 (mg/dl) | 103.9 | 79.0-152.0 | Hyaline casts (cells/μl) | 0 | 0 |
| Complement C4 (mg/dl) | 33.8 | 16.0-38.0 |  |  |  |
| Cystatin C (mg/L) | 2.73 | 0.54-1.54 |  |  |  |
| β2-microglobulin (mg/L) | 6.38 | 1.00-2.30 |  |  |  |
| Anti-streptolysin O (IU/ml) | 90 | ＜200 |  |  |  |
| ESR (mm/h) | 14 | ＜15 |  |  |  |
| Rheumatoid factor(IU/ml) | 2 | ＜14 |  |  |  |
| anti-Glomerular Basement Membrane-IgG(RU/mL) | ＜20 | ＜20 |  |  |  |

Table S3 Urine-sensitive kidney function panel

| **Items** | **Results** | **Ref** |
| --- | --- | --- |
| Urine creatinine (mmol/L) | 4.27 | 3.90-27.10 |
| Urine N-acetyl-β-D-glucosaminidase (U/L) | ＜2.5 | ＜12.0 |
| Urine β2-microglobulin (μg/L) | 868 | ≤300 |
| Urine microalbumin (mg/L) | 18.11 | ＜30.00 |
| Urine immunoglobulin IgG (mg/L) | 2.7 | ＜9.60 |
| Urine retinol-binding protein (mg/L) | 0.37 | ≤0.70 |
| Urine transferrin (mg/L) | 1.05 | ＜2.00 |
| Urine NAG/urine creatinine (U/mmolCr) | 0.37 | ≤1.30 |
| Urine microalbumin/urine creatinine (mg/gCr) | 37.53 | ＜30.00 |

Table S4 Vasculitis-Related Nephritis Test Panel

| **Items** | **Results** | **Ref** |
| --- | --- | --- |
| cANCA (anti-neutrophil cytoplasmic antibodies) | Negative | Negative |
| pANCA (anti-neutrophil cytoplasmic antibodies) | Negative | Negative |
| Anti-neutrophil cytoplasmic proteinase 3 antibody (RU/mL) | ＜20 | ＜20 |
| Anti-neutrophil cytoplasmic myeloperoxidase (RU/mL) | ＜20 | ＜20 |

## Supplementary Figure S1: The detailed clinical visit flowchart
